# The Effectiveness of Anti-Interleukin-17A Treatment for Pityriasis Rubra Pilaris: A Systematic Review

**DOI:** 10.7759/cureus.41125

**Published:** 2023-06-29

**Authors:** Mohammad Abduljawad, Thamer H Alsharif, Amin G Gronfula, Talah K Magadmi, Lujain I Khayat, Sarah M Fageeh, Abdulqader A Almuallim, Mohammad Ayman Mohammad, Abdullah Albadri

**Affiliations:** 1 Dermatology, King Fahad General Hospital, Jeddah, SAU; 2 Neurosurgery, Royal College of Surgeons in Ireland, Dublin, IRL; 3 Orthopaedic Surgery, Royal College of Surgeons in Ireland, Dublin, IRL; 4 Medicine and Surgery, King Abdulaziz University, Jeddah, SAU; 5 Medicine and Surgery, Taibah University, Medina, SAU; 6 Medicine, Faculty of Medicine, Umm Al-Qura University, Makkah, SAU; 7 Internal Medicine, Umm Al-Qura University, Makkah, SAU; 8 Oncology, Princess Norah Oncology Centre, King Abdulaziz Medical City, Jeddah, SAU

**Keywords:** anti-interleukin-17a, ixekizumab, pityriasis rubra pilaris, prp, • secukinumab

## Abstract

Anti-interleukin-17A (anti-IL-17A) therapy has been increasingly employed as a treatment option for pityriasis rubra pilaris (PRP). In this study, we reviewed all available studies on this topic in the literature to evaluate the efficacy and safety of anti-IL-17A. Our main objective was to assess the current evidence on the efficacy and safety of anti-IL-17A therapy in the management of PRP. We searched for relevant articles on PubMed, MEDLINE, Ovid, Embase, and the Web of Science electronic databases from inception until 2022. Our inclusion criteria were as follows: randomized controlled trials (RCTs), quasi-randomized trials, or prospective observational studies that include PRP patients treated with biological treatments; studies that report clinical outcomes; and studies that compare the treatment modalities, including anti-IL-17, in the English language.

A total of 19 articles involving 77 cases were reviewed after applying the inclusion criteria and removing duplicates. We found that type 1 PRP was the most common condition irrespective of gender, and the trunk was the most affected area. The study showed that IL-17 inhibitors had a significant impact on the patients. However, higher-level studies are required to further evaluate the therapeutic and safety effects of the treatment.

## Introduction and background

Pityriasis rubra pilaris (PRP) is a rare form of dermatosis; it is characterized by a cluster of hyperkeratotic follicular papules, erythematous scaly patches coalescing to form orange-red plaques with the characteristic of "island of sparing", and keratoderma that is present on the palms and soles [1]. Due to its rarity, the exact incidence of the disease is not well established. While it usually affects people aged above 50 years, people of all ages, races, and genders can be affected. The condition can be categorized into six clinical subtypes based on Griffith’s classification. These subtypes are classified depending on the involvement of either ichthyosis or scleroderma clinical features, the extent of the disease, the age of onset of the disease, and the disease prognosis. The most common subtype is type I, which accounts for 55% of all cases; it is usually acute in onset and appears in the areas of the face and neck, later spreading further down to the trunk, arms, and legs. It has been observed that patients can also develop brownish-yellow discoloration of the nails with subungual hyperkeratosis and longitudinal ridging [2]. The diagnosis of PRP is dependent on the above-mentioned clinical features and histopathology, taking into account certain characteristics such as the checkerboard pattern of alternating orthokeratosis and parakeratosis, localized solitary or diffused hypergranulosis, irregular acanthosis, widened supra-papillary plates, scanty superficial perivascular lymphohistiocytic infiltrates occupying the dermis, follicular plugging, as well as less-classified morphologies such as the lichenoid infiltrate, dermal eosinophilia, and acantholysis [2].

Regarding the treatment options for PRP, topical corticosteroids have been the mainstay of treatment, having a significant impact, especially in the pediatric age group, those with mild disease, and certain specific races. Also, based on several recent case series, systemic retinoids have had an impact on the treatment of PRP by acting on the intranuclear retinoic acid receptors. Another additive or alternative to systemic retinoids is methotrexate [3]. The use of biologics in PRP has shown promising results, including the use of tumor necrosis factor (TNF)-alpha inhibitors like infliximab, etanercept, or adalimumab. Some research has observed treatment success with one of these treatments despite prior treatment failures with agents from the same class [4]. For the past few years, the off-label use of the human monoclonal antibody secukinumab, which binds and neutralizes interleukin 17A (IL-17A), has shown favorable outcomes in patients with PRP [5].

Recently, there has been growing interest in the use of anti-IL-17A therapy for the treatment of PRP. In light of this, this systematic review aims to evaluate the current evidence on the efficacy and safety of anti-IL-17A therapy in the management of PRP. By synthesizing the available data, we hope to provide clinicians with a better understanding of this treatment option and guide future research efforts.

## Review

Material and methods

Data Sources and Search Strategy

A literature search was conducted using PubMed, MEDLINE Ovid, Embase, and Web of Science electronic databases. from inception until March 2022. All relevant articles were identified using the following keywords: "Pityriasis Rubra Pilaris," "Anti-Interleukin-17A," "Secukinumab," "Cosentyx," "biological treatment," and "monoclonal antibodies." The systematic review followed the Preferred Reporting Items for Systematic Reviewers and Meta-analysis (PRISMA) guidelines [6].

Eligibility Criteria

The following criteria were used to select eligible studies: randomized controlled trials (RCTs), quasi-randomized trials, or prospective observational studies that include PRP patients treated with biological agents; studies that report clinical outcomes; studies that compare PRP treatment modalities, including anti-IL-17; and studies in English. The exclusion criteria were as follows: systematic reviews; studies using animal models; studies not in English; and studies that do not report clinical outcomes.

Study Selection and Data Extraction

Two reviewers (TM) and (LA) independently eliminated studies by reading their titles and abstracts, eliminated duplicates, and then confirmed as to which studies were eligible for this systematic review in accordance with the inclusion and exclusion criteria. A third reviewer (MA) resolved any disagreement between the two reviewers. Using data collection forms on a Google Sheet, TM and LA independently collected the data from the selected papers. The following variables were collected: author/year/country, age in years, gender, PRP type, clinical features, primary treatment received, IL-17 treatment outcome, and side effects. All variables were collected on a Microsoft Excel sheet. For data analysis and the creation of figures, Microsoft Excel Version 16.71 was used.

Results

Search Results and Study Selection 

**Figure 1:**
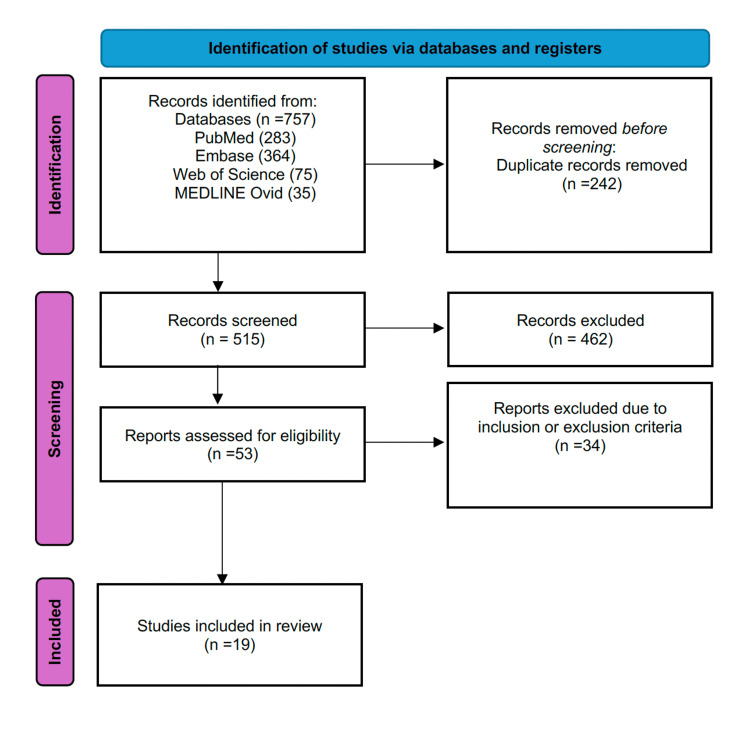
PRISMA flow diagram PRISMA: Preferred Reporting Items for Systematic Reviews and Meta-Analyses

Our preliminary search of the databases elicited a total of 757 articles, with 283 in PubMed, 364 in Embase, 75 in Web of Science, and 35 in MEDLINE Ovid. We then used EndNote Reference Manager (Version 20.5) to identify and eliminate duplicate articles, resulting in a total of 515 unique articles. After screening the titles and abstracts of these articles based on predetermined inclusion and exclusion criteria, we were left with 53 articles for full-text screening, while 462 articles were excluded. Ultimately, our systematic review included 19 articles, as indicated in the PRISMA flow diagram (Figure 1).

Study Characteristics 

Table 1 summarizes of characteristics of the studies and responses to anti-IL-17 administration based on the clinical outcomes. This review comprised a total of 77 cases, with a higher proportion of male patients (55 males and 22 females). The average age of these patients was 37.35 ± 28.58 years: range: 1-83 years (37.71 ± 28.81 years for males and 35.11 ± 28.56 years for females). Based on the analyses, type I PRP was the most common type of PRP identified (n=31 cases), followed by type II (n=12 cases), type III (n=5 cases), and type IV (n=3 cases). However, the type of PRP was not identified in 26 cases.

**Table 1:** Summary of the studies’ characteristics PRP: pityriasis rubra pilaris; M: male; F: female

Author	N	Age (years)	PRP type	Treatment	Outcome
Gauci et al. [1]	1F	33	Type III	Secukinumab	Complete response
Schuster et al. [5]	1M	67	NA	Secukinumab	Complete response
Strunck et al. [7]	8M, 3F	Mean age: 47.3	NA	Ixekizumab	Partial response
Penalba-Torres et al. [8]	1F	83	Type I	Ixekizumab	Complete response
Albela et al. [9]	1F	4	Type IV	Secukinumab	Almost complete
Chastagnr et al. [10]	1F	48	NA	Ixekizumab	Complete response
Kevric et al. [11]	1F	54	Type III	Secukinumab	Complete response
Haynes et al. [12]	8M, 4F	Mean age: 54	6 type I, 5 type II, 1 type III	Ixekizumab	Almost complete
Wain et al. [13]	3M	Mean age: 50	2 type I and 1 type II	Secukinumab	1 complete response, 1 partial response, and 1 worsening
Xu et al. [14]	1M	3	Type III	Secukinumab	Almost complete response
Boudreaux et al. [15]	11M, 1F	Mean age: 70	NA	Secukinumab	Complete response
Papini et al. [16]	6M, 2F	Mean age: 59.3	6 type I, type II	Secukinumab	4 complete responses, 3 partial responses, and 1 almost complete response
Napolitano et al. [17]	3M, 2F	Mean age: 43.2	4 type I, 1 type II	Secukinumab	4 complete responses, 1 partial response
Matsuda et al. [18]	1M	67	Type I	Secukinumab	Complete response
Liu and Wang [19]	1M	67	Type IV	Ixekizumab	Complete response
Liang et al. [20]	9M, 4F	Mean age: 56.30	9 type I, 2 type II, 1 type III, 1 type IV	Secukinumab	Complete response
Bonomo and Levitt [21]	1M, 1F	Mean age: 67	Type I, type II	Secukinumab	Complete response, worsening
Heibel and Heibel [22]	1M	63	Type I	Ixekizumab	Complete response
Hanfstingl et al. [23]	1M	68	NA	Ixekizumab	Complete response

As shown in Table 2, we focused on the identification of different types of PRP between the genders. We discovered that type 1 was the most common among both genders. However, males outnumbered females, with a ratio of 25:6. In a couple of studies, the type of PRP was not mentioned for a total of 26 patients. Type 2 accounted for the same number of cases among both genders, with six cases in each gender. 

**Table 2:** Demographic data of PRP patients and the associated PRP types PRP: pityriasis rubra pilaris

Gender	Type I	Type II	Type III	Type IV	NA	Grand total
Male	25	6	2	1	21	55
Female	6	6	3	2	5	22
Total	31	12	5	3	26	77

As presented in Table 3, we also focused on the affected areas among the patients. The trunk was the most commonly affected area (n=23 cases), followed by the palms (n=16 cases). The knees, eyes, and back were the least affected areas, with one reported case involving each of these areas. In most patients, the primary treatments used were acitretin, methotrexate, infliximab, and isotretinoin. The aim of our study was to examine the results and compare the effectiveness of the above-mentioned primary treatments with that of the IL-17 inhibitors. As we can see from the results, IL-17 inhibitors had a significant impact, and their effectiveness was high. Of the 19 studies, 12 discussed the use of secukinumab as a treatment method, while seven mentioned ixekizumab.

**Table 3:** Most commonly affected body area in the patients

Affected area	Number of cases
Trunk	23
Palms	16
Face	11
Scalp	9
Soles, neck	6
Nails	5
Arm	4
Chest, legs	3
Head, buttocks	2
Most of the body	2
Back, eyes, knees	1

Outcomes With Secukinumab and Ixekizumab

Among the total of 77 patients from 19 studies, 63% were treated with secukinumab, which showed an almost 90% complete improvement among all types of PRP. Six cases of type III and IV had a 100% complete response; 21/23 patients with type 1 experienced complete remission. As shown in Table 4, secukinumab showed a more significant response in types 1, 3, and 4. Patients with type 2 PRP had only a 57% response to secukinumab; 13 patients with an unknown type of PRP had 100% remission. The dose administered was 300 mg weekly for five weeks, then monthly, and the average duration was six months, as shown in Table 4. Also, ixekizumab showed a significant impact on type 1 PRP with complete remission among all eight patients who had received the drug. Complete remission was also noted among patients taking ixekizumab for types 2, 3, and 4. Of note, 13 patients with an unknown PRP type did not show a good response to ixekizumab, with only 18% responding in comparison to 100% in the other groups. The loading dose was 160 mg per week. 

**Table 4:** Treatment of PRP and its outcomes PRP: pityriasis rubra pilaris

PRP type	Secukinumab	Outcome	Ixekizumab	Outcome
Type I	23	91% complete response	8	100% complete response
Type II	7	57% complete response	5	100% complete response
Type III	4	100% complete response	1	100% complete response
Type IV	2	100% complete response	1	100% complete response
NA	13	100% complete response	13	18% complete response

Discussion 

There is significant room for improvement in available treatments for PRP; the use of IL-17 in the treatment of patients with PRP is relatively new. We conducted this systemic review to assess the effectiveness of IL-17 inhibitors in the treatment of PRP. Although retinoids were always the mainstay and primary treatment for all patients suffering from PRP, in recent years, biological treatments have shown a significant impact on patients with psoriasis [24]. The substantial side-effect burden associated with the use of previous treatment modalities in comparison to their effectiveness raised the need for introducing new treatment regimens.

Clinical trials have been conducted to examine the effectiveness of biologics in the management of PRP [25,26]. In our study, we gathered data that compared different types of PRP from various studies, in which different types of treatment modalities were used. The research focused on the improvement in the Psoriasis Area and Severity Index (PASI) score among patients taking IL-17 inhibitors. The improvement in the PASI score was measured on a weekly basis. Patients taking IL-17 inhibitors showed significant improvement when compared to the retinoid population. Many physicians have started adding IL-17 inhibitors to their treatment plans for PRP; however, the safety concerns need to be addressed.

The recommended dosages are as follows: ixekizumab subcutaneous (SC) - 160 mg once (week 0; administered as two separate 80 mg injections), followed by 80 mg every two weeks (at weeks two, four, six, eight, 10, and 12) in six doses, and then 80 mg every four weeks. Secukinumab SC: 300 mg weekly for five weeks, and then every four weeks.

The findings of this article should be interpreted cautiously due to several limitations. The review only included case reports and case series with small sample sizes. Additionally, RCTs were lacking, and treatments with biologics, such as ixekizumab and secukinumab, were only reported in a few patients, which may introduce bias regarding their efficacy due to selective reporting of positive outcomes. Furthermore, up to 80% of type 1 PRP cases are known to resolve within three years of symptom onset without treatment, making it unclear whether symptom resolution was due to biological treatment or the natural course of the disease. The relapsing and self-limited nature of PRP also diminishes the significance of treatment outcomes. Finally, limited information was available regarding previous treatments and concomitant therapies, which makes it challenging to determine the therapeutic role of specific biologics in treating PRP [27,28].

The available data on the safety and efficacy of biologics in treating PRP are limited due to the low methodological quality, small sample sizes, and heterogeneity in treatment regimens, which restricts the applicability of these findings to other types of PRP. Additional research is needed to determine the effectiveness of specific biologic therapies in treating treatment-resistant PRP with greater accuracy. Due to the limited evidence available, we cannot recommend a treatment algorithm at this time, but a practical approach would be to start with retinoids, preferably acitretin, as the first-line therapy, followed by immunosuppressive treatments like MTX as the second-line therapy, and biologics as the third-line therapy. The results from case studies and case series using biologics to treat PRP are promising, but RCTs are necessary to evaluate their therapeutic efficacy and safety in a more precise manner. In conclusion, while initiating biologic monotherapy alone appears to be effective, further research is needed to fully evaluate the therapeutic potential of individual biologics [29,30].

## Conclusions

Biological treatment with anti-IL-17A inhibitors, such as secukinumab and ixekizumab, showed a significant impact on PRP patients based on the data gathered from case reports and case series. However, higher-level studies and larger sample sizes are required to more comprehensively evaluate the therapeutic efficacy and safety of this treatment method.

## References

[1] Gauci ML, Jachiet M, Gottlieb J, Madeleine-Chambrin I, Rybojad M, Bagot M, Bouaziz JD (2016). Successful treatment of type II pityriasis rubra pilaris with secukinumab. JAAD Case Rep.

[2] Wang D, Chong VC, Chong WS, Oon HH (2018). A review on pityriasis rubra pilaris. Am J Clin Dermatol.

[3] Dicken CH (1994). Treatment of classic pityriasis rubra pilaris. J Am Acad Dermatol.

[4] Vergilis-Kalner IJ, Mann DJ, Wasserman J, Petronic-Rosic V, Tsoukas MM (2009). Pityriasis rubra pilaris sensitive to narrow band-ultraviolet B light therapy. J Drugs Dermatol.

[5] Schuster D, Pfister-Wartha A, Bruckner-Tuderman L, Schempp CM (2016). Successful treatment of refractory pityriasis rubra pilaris with secukinumab. JAMA Dermatol.

[6] Page MJ, McKenzie JE, Bossuyt PM (2021). The PRISMA 2020 statement: an updated guideline for reporting systematic reviews. Int J Surg.

[7] Strunck JL, Cutler B, Rajpal B (2022). Pityriasis rubra pilaris response to IL-17A inhibition is associated with IL-17C and CCL20 protein levels. J Invest Dermatol.

[8] Penalba-Torres M, Pinilla-Martín B, Aragón-Miguel R, Velasco-Tamariz V, Rivera-Díaz R (2020). Successful treatment of resistant pityriasis rubra pilaris with ixekizumab. Dermatol Ther.

[9] Albela H, Lee WQ, Nordin SN, Gopinathan LP, A Ramli MN, Leong KF (2022). Juvenile pityriasis rubra pilaris in a 4-year-old child treated successfully with secukinumab. Indian J Dermatol.

[10] Chastagner M, Hoelt P, Kanitakis J, Jullien D, Villani AP (2019). Successful treatment of TNFα inhibitor-resistant pityriasis rubra pilaris with ixekizumab and acitretin. Eur J Dermatol.

[11] Kevric I, Sluzevich J (2016). Response of persistent juvenile pityriasis rubra pilaris to secukinumab. JAAD Case Rep.

[12] Haynes D, Strunck JL, Topham CA (2020). Evaluation of ixekizumab treatment for patients with pityriasis rubra pilaris: a single-arm trial. JAMA Dermatol.

[13] Wain T, Choy B, Satchell AC, Woods JA, Frew JW (2018). Secukinumab in pityriasis rubra pilaris: a case series demonstrating variable response and the need for minimal clinical datasets. JAAD Case Rep.

[14] Xu YH, Dong DD, Lin YF, Wang Q, Huang LM, Shi JQ (2022). Successful treatment of severe pityriasis rubra pilaris with secukinumab in a 3-year-old boy. Clin Exp Dermatol.

[15] Boudreaux BW, Pincelli TP, Bhullar PK (2022). Secukinumab for the treatment of adult-onset pityriasis rubra pilaris: a single-arm clinical trial with transcriptomic analysis. Br J Dermatol.

[16] Papini M, Russo A, Natalini Y, Troiani L, Cassiani L (2021). Secukinumab is an effective and safe treatment for refractory pityriasis rubra pilaris. Ital J Dermatol Venerol.

[17] Napolitano M, Lembo L, Fania L, Abeni D, Didona D, Didona B (2018). Ustekinumab treatment of pityriasis rubra pilaris: a report of five cases. J Dermatol.

[18] Matsuda T, Yamazaki F, Ueda-Hayakawa I, Kambe N, Okamoto H (2019). Case of pityriasis rubra pilaris progressed to generalized erythroderma following blockade of interleukin-17A, but improved after blockade of interleukin-12/23 p40. J Dermatol.

[19] Liu YT, Wang SS (2022). Ixekizumab successfully treated severe pityriasis rubra pilaris after COVID-19 vaccination. Skin Health Dis.

[20] Liang JY, Ye RX, Tian X, Zhang SQ, Zhang XB (2020). Secukinumab monotherapy successfully treated severe refractory type V (atypical juvenile) pityriasis rubra pilaris: a case report and literature review. Dermatol Ther.

[21] Bonomo L, Levitt JO (2018). Secukinumab emerges as a rapidly effective therapy for pityriasis rubra pilaris. Cutis.

[22] Heibel MD, Heibel HD (2018). Successful treatment of type I pityriasis rubra pilaris with ixekizumab. JAAD Case Rep.

[23] Hanfstingl K, Pekar-Lukacs A, Motz R, Guenova E, Hoetzenecker W (2018). Successful treatment of pityriasis rubra pilaris with ixekizumab. Case Rep Dermatol.

[24] Ross NA, Chung HJ, Li Q, Andrews JP, Keller MS, Uitto J (2016). Epidemiologic, clinicopathologic, diagnostic, and management challenges of pityriasis rubra pilaris: a case series of 100 patients. JAMA Dermatol.

[25] Blanchet-Bardon C, Nazzaro V, Rognin C, Geiger JM, Puissant A (1991). Acitretin in the treatment of severe disorders of keratinization. Results of an open study. J Am Acad Dermatol.

[26] Aragón-Miguel R, Prieto-Barrios M, Calleja-Algarra A, Velasco-Tamariz V, Andres-Lencina JJ, Ortiz-Romero P, Monsálvez-Honrubia V (2018). Refractory pityriasis rubra pilaris with good response after treatment with ustekinumab. J Dtsch Dermatol Ges.

[27] Borok M, Lowe NJ (1990). Pityriasis rubra pilaris: further observations of systemic retinoid therapy. J Am Acad Dermatol.

[28] Murad MH, Sultan S, Haffar S, Bazerbachi F (2018). Methodological quality and synthesis of case series and case reports. BMJ Evid Based Med.

[29] Ivanova K, Itin P, Haeusermann P (2012). Pityriasis rubra pilaris: treatment with biologics - a new promising therapy?. Dermatology.

[30] Roenneberg S, Biedermann T (2018). Pityriasis rubra pilaris: algorithms for diagnosis and treatment. J Eur Acad Dermatol Venereol.

