# Correction: The Effectiveness of Anti-Interleukin-17A Treatment for Pityriasis Rubra Pilaris: A Systematic Review

**DOI:** 10.7759/cureus.c348

**Published:** 2025-10-07

**Authors:** Mohammad Abduljawad, Thamer H Alsharif, Amin G Gronfula, Talah K Magadmi, Lujain I Khayat, Sarah M Fageeh, Abdulqader A Almuallim, Mohammad Ayman Mohammad, Abdullah Albadri

**Affiliations:** 1 Dermatology, King Fahad General Hospital, Jeddah, SAU; 2 Neurosurgery, Royal College of Surgeons in Ireland, Dublin, IRL; 3 Orthopaedic Surgery, Royal College of Surgeons in Ireland, Dublin, IRL; 4 Medicine and Surgery, King Abdulaziz University, Jeddah, SAU; 5 Medicine and Surgery, Taibah University, Medina, SAU; 6 Medicine, Faculty of Medicine, Umm Al-Qura University, Makkah, SAU; 7 Internal Medicine, Umm Al-Qura University, Makkah, SAU; 8 Oncology, Princess Norah Oncology Centre, King Abdulaziz Medical City, Jeddah, SAU

This article has been corrected to fix the following typo: 'Reports excluded due to inclusion or exclusion criteria' has been corrected from 33 to 34.

